# Development of Lipidic Nanoplatform for Intra-Oral Delivery of Chlorhexidine: Characterization, Biocompatibility, and Assessment of Depth of Penetration in Extracted Human Teeth

**DOI:** 10.3390/nano12193372

**Published:** 2022-09-27

**Authors:** Krishnaraj Somyaji Shirur, Bharath Singh Padya, Abhijeet Pandey, Manasa Manjunath Hegde, Aparna I. Narayan, Bola Sadashiva Satish Rao, Varadaraj G. Bhat, Srinivas Mutalik

**Affiliations:** 1 Department of Conservative Dentistry and Endodontics, Manipal College of Dental Sciences Manipal, Manipal Academy of Higher Education, Manipal 576104, Karnataka State, India; 2 Department of Pharmaceutics, Manipal College of Pharmaceutical Sciences, Manipal Academy of Higher Education, Manipal 576104, Karnataka State, India; 3 Department of Radiation Biology & Toxicology, Manipal School of Life Sciences, Manipal Academy of Higher Education, Manipal 576104, Karnataka State, India; 4 Department of Prosthodontics and Crown and Bridge, Manipal College of Dental Sciences Manipal, Manipal Academy of Higher Education, Manipal 576104, Karnataka State, India; 5 Department of Pharmaceutical Chemistry, Manipal College of Pharmaceutical Sciences, Manipal Academy of Higher Education, Manipal 576104, Karnataka State, India

**Keywords:** nano-liposomes, chlorhexidine, confocal microscopy, cytotoxicity, depth of penetration

## Abstract

Microorganisms are the major cause for the failure of root canal treatment, due to the penetration ability within the root anatomy. However, irrigation regimens have at times failed due to the biofilm mode of bacterial growth. Liposomes are vesicular structures of the phospholipids which might help in better penetration efficiency into dentinal tubules and in increasing the antibacterial efficacy. Methods: In the present work, chlorhexidine liposomes were formulated. Liposomal chlorhexidine was characterized by size, zeta potential, and cryo-electron microscope (Cryo-EM). Twenty-one single-rooted premolars were extracted and irrigated with liposomal chlorhexidine and 2% chlorhexidine solution to evaluate the depth of penetration. In vitro cytotoxicity study was performed for liposomal chlorhexidine on the L929 mouse fibroblast cell line. Results: The average particle size of liposomes ranged from 48 ± 4.52 nm to 223 ± 3.63 nm with a polydispersity index value of <0.4. Cryo-EM microscopic images showed spherical vesicular structures. Depth of penetration of liposomal chlorhexidine was higher in the coronal, middle, and apical thirds of roots compared with plain chlorhexidine in human extracted teeth when observed under the confocal laser scanning microscope. The pure drug exhibited a cytotoxic concentration at which 50% of the cells are dead after a drug exposure (IC_50_) value of 12.32 ± 3.65 µg/mL and 29.04 ± 2.14 µg/mL (on L929 and 3T3 cells, respectively) and liposomal chlorhexidine exhibited an IC_50_ value of 37.9 ± 1.05 µg/mL and 85.24 ± 3.22 µg/mL (on L929 and 3T3 cells, respectively). **Discussion:** Antimicrobial analysis showed a decrease in colony counts of bacteria when treated with liposomal chlorhexidine compared with 2% chlorhexidine solution. Nano-liposomal novel chlorhexidine was less cytotoxic when treated on mouse fibroblast L929 cells and more effective as an antimicrobial agent along with higher penetration ability.

## 1. Introduction

Microorganisms are one of the vital factors responsible for the failure of endodontic treatment. In the development and perpetuation of pulpal and periapical diseases, bacteria play an essential role which has been demonstrated in human models and animal studies [1]. Chlorhexidine digluconate (CHX) is a potential drug molecule for endodontic infections. CHX interacts with phospholipids and lipopolysaccharides on the bacterial cell membrane and then enters the bacterium through the active or passive transportation mechanism. CHX is a well-known dental antiseptic and a non-specific matrix metalloproteinase (MMP) inhibitor that hinders the comprehensive actions of collagenolytic or gelatinolytic enzymes, such as MMP-2, MMP-8, and MMP-9 present in dentin [2]. CHX has the property of substantivity due to which it binds to hard tissue and retains its potential antimicrobial efficacy. This property has made CHX a popular and effective drug candidate in these incidences [3]. This drug can alter the cell’s osmotic equilibrium as the microbial cell wall is negatively charged and extra molecular complexes of the bacterial cell can strongly bind to cationic moieties, such as CHX [4]. Some microbes at times represent potential new bacterial phylotypes [5]. Irrigating solutions, such as sodium hypochlorite, when extruded into peri-apical tissue can cause severe pain and also can have the cytotoxic potential [6]. Although CHX has widely been considered as a standard drug for endodontic applications, microorganisms are generally observed even after its use, and it has been attributed to a lack of penetrating efficiency of the drug into dentinal tubules [3]. The presence of bacteria and their penetration depth in various surfaces of dentin and into dentinal tubules have been justified [3]. Peters et al. reported that due to the bacterial growth on cemental surfaces in 62% and 24% of cases, 50,000 CFU/g were seen on the cemental surface of root cementum [7]. In this report, the chlorhexidine nanocapsule drug delivery approach was successfully applied to the resin dentin interface [8]. Nano-liposomes are sub-microscopic nano-sized lipidic vesicles that can encapsulate different types of drugs. Currently, there are scarcely any reports available on the evaluation of the nano-liposomes loaded with CHX. Fabrication of liposomes is one of the approaches to improve the efficacy of CHX and to overcome the drawbacks.

Liposomes are artificial vesicles that are spherical in shape, created from non-toxic phospholipids and cholesterol. Liposomes are potential systems for drug delivery due to their hydrophobic and hydrophilic characters and size. Method of preparation, composition of lipid, size, and surface charge considerably change the liposomal properties. Rigidity or fluidity is determined by the choice or selection of the bilayer component and the bilayer charges. The unsaturated phosphatidylcholine species occurring from natural sources provide a less stable and more increased permeable bilayer. However, saturated phospholipids with long acyl chains form rather impermeable and rigid bilayers. Nanoencapsulation can further lengthen the delivery period and helps in better medicament release, which increases the bactericidal effect of the drug in dentinal tissues. The size of liposome can range from very small (0.025 μm) to large (2.5 μm) vesicles. The liposomal formulation can be a single layer or bilayered. Drug encapsulation efficiency is dependent on the size and number of bilayers in the liposomes [9,10].

Considering the advantages of nano-liposomes, such as biocompatibility, non-toxic nature, and nano-size, an attempt has been made to prepare and evaluate liposomal CHX as an irrigating solution in endodontic applications in the present study. With nano-liposomal CHX as an irrigant, it is expected that the liposomal CHX irrigant penetrates deep into the dentinal tubules, which would further reach towards the penetrated bacteria. An extended contact time or stronger binding of nano-form of the antibiotic with biofilm can be possible with this approach [11,12]. CHX at 0.12% and 2% concentrations is used for mouth wash and endodontic disinfection, respectively [13]. Significance of this study lies in developing liposomal CHX and its use as an irrigant may help in penetrating CHX and disrupting the biofilm matrix in the oral mucosa and tooth to a greater extent. Its application in endodontics as an irrigant might aid in deeper penetration into dentinal tubules leading to more effective eradication of bacteria. The aim of this study was to develop the nano-liposomal CHX and, to characterize (size, zeta potential, polydispersity index, and surface topography) and evaluate them with respect to the drug release study, cytotoxicity in L929 mouse fibroblasts, antibacterial efficacy against *Fusobacerium nucleatum*, *Staphylococcus aureus*, and *Streptococcus mutans*, and the depth of penetration into dentinal tubules.

## 2. Materials and Methods

### 2.1. Materials

Chlorhexidine digluconate (CHX; 20%) was purchased from Unilab Pharmaceuticals, Mumbai, India. Hydrogenated soy-phosphatidylcholine (HSPC) and cholesterol were supplied by Lipoid, Newark, NJ, USA and HiMedia, Mumbai, India, respectively. Tween 80 and chloroform were purchased from Finar Chemicals, Ahmedabad, Gujarat, India. The rest of the chemicals used for the preparation of liposomes were of analytical/reagent grade.

L929 and 3T3 cells were purchased from the National Centre for Cell Science (NCCS), Pune and the cell line study was carried out at Radiant Research Services Pvt. Ltd., Bangalore, India. This study was inspected regularly according to the Standard Operating Procedure of the test facility’s Quality Assurance Unit.

### 2.2. Preparation of Liposomal CHX

The CHX solution was lyophilized and used in the powder form. Thin-film hydration technique was used for the preparation of liposomes according to the previous work reported by our group [14,15].

Briefly, HSPC and cholesterol (90:10; 100 mg of total lipids) were dissolved in 10 mL of chloroform in a round-bottomed flask. The organic solvent was evaporated in a rotavapor under vacuum to obtain a thin film. The dried thin film was hydrated by a phosphate buffer solution of pH 7.4 containing 30 mg of CHX. After hydration, the dispersion was subjected to sonication using a probe sonicator (Model-VCX750, Sonics & Materials, Inc., Newtown, CT, USA) for 20 min at 40% amplitude (750 watt) and 6 s pulse. The dispersion was further subjected to high-speed centrifugation (at 22,000 rpm and 4 °C for 45 min) to separate the free drug. The liposomal pellet was re-dispersed in 5 mL of water and stored in a refrigerator. A similar liposomal formulation was prepared by incorporating rhodamine B dye (1% *w/v* solution) in the chloroform lipid solution to visualize under a confocal laser scanning microscope. The composition of different batches of liposomes is provided in Table 1. The control sample of liposomal formulation, without the drug, was also prepared.

### 2.3. Characterization of Liposomal CHX

#### 2.3.1. Determination of Particle Size, Poly-Dispersibility Index (PDI), and Zeta Potential

The formulated liposomal CHX was analyzed for particle size, PDI, as well as zeta potential using Zeta Sizer (NanoZS, Malvern Instruments, UK). The particle size of the liposomal formulations was determined by the dynamic light scattering (DLS) method. Liposomes were irradiated with a laser to the middle of the cell region at a fixed detection array of 90 ranges and variations in the intensities of the dispersed light were analyzed. Later, the obtained results were considered as an average of 10 measurements. In the presence of electric field, the electrophoretic mobility of the particles takes place, which is the basis of the determination of zeta potential, and is determined by laser doppler velocimetry (LDV) and phase analysis light scattering (PALS) techniques [16,17].

#### 2.3.2. Determination of Encapsulation Efficiency

The entrapment efficiency of CHX liposomes was determined using the previously reported method. In brief, liposomes were dissolved in absolute alcohol (5 mL) with the aid of water-bath sonication for 10 min along with Triton X. The concentration of CHX was determined spectrophotometrically in the resulting solution at 254 nm using an UV-visible spectrophotometer (UV-1700E, Shimadzu, Kyoto, Japan) in triplicate. For the estimation of CHX, 254 nm was selected as λmax, which was determined by the scanning CHX solution in UV/Vis spectrophotometer. The figures related to this experiment are provided in the Supplemented Material (I. Selection of 254 nm as λmax for the estimation of CHX). The UV spectroscopic method was optimized at 254 nm with the blank solution containing the blank liposomal solution (containing only the excipients used for preparing the liposomes). Therefore, the presence of ingredients of broken liposomes did not affect the accuracy of the estimation. Corresponding UV/Vis spectroscopic scans for plain CHX solution, blank liposomes, and CHX liposomes are provided in the Supplemented Material (Figures S1–S3). The efficiency of encapsulation expressed as the percentage of entanglement was determined through the following relationship [18]:

(1) Entrapment efficiency (%)=(Total drug−free drug)/(Total drug)×100

#### 2.3.3. Surface Morphology

The surface morphology of the prepared liposomal CHX was studied using the cryo-electron microscope (Gatan Alto 2500 Cryo Transfer System, Pleasanton, CA, USA). The samples of liposomal CHX were dropped on a copper grid and then analyzed under EM at various magnifications and power. The images were captured and later analyzed.

### 2.4. Fourier-Transform Infrared Spectroscopy (FTIR)

The FTIR spectra of plain CHX, physical mixture of CHX + excipients, and lyophilized CHX liposomes (Batch 3) were analyzed to assess the possible interactions. To evaluate the samples using FTIR, the samples were mixed with KBr (1:1 *w*/*w*) and the spectra were recorded in the region of 4000–400 cm^−1^ using FTIR8300 (Shimadzu, Kyoto, Japan).

### 2.5. In Vitro Drug Release Study

In vitro drug release from the liposomal formulation was carried out using the Electrolab TDT-08L dissolution tester (USP). Phosphate buffer (pH 6.8) was used as a dissolution medium (as oral cavity pH ranges from 6.7–7.3) for assessing the drug release. The samples of dissolution medium were collected at specific time points (1 mL of the dissolution medium) and replaced with a fresh buffer solution. The collected samples were then analyzed using UV/Visible spectrophotometer and the drug release was estimated.

### 2.6. Preparation of the Teeth for Experimentation

Premolar single-rooted mandibular teeth were collected after extraction for orthodontic reasons. They were cleaned thoroughly and stored in peroxide solution. This study measures the depth of penetration of two irrigants. Before the study, the sample size was derived by assuming that the mean difference between the depth of penetration obtained by two different irrigants may be 1 µm, and thus the “effect size” was assumed to be 1 µm. The confidence interval indicates that there might be variation of >1 µm or <1 µm in order that this variation may be 95%. Based on this assumption, a total sample size of 16 teeth was estimated by assuming the effect size of 1 µm at 95% confidence interval and 80% power.

However, the minimum sample size estimated was 16 and a total of 45 teeth were collected. Approval from the Institutional Ethical Committee was obtained (Institutional Ethical Committee Approval No.: 27/2019, Kasturba Medical College and Kasturba Hospital, Manipal) for this procedure. The informed consent from the patients was not obtained as the anonymized extracted teeth were used in this study. Out of the 45 teeth collected, some of the teeth were discarded due to irregularities in the teeth. Finally, the number of teeth used for the study was 21. All the teeth were de-coronated at the cemento-enamel junction and were standardized to uniform length with the help of a diamond disc. Working length was arbitrarily 10 mm for all the selected teeth. Biomechanical preparation was performed with ProTaper rotary files up to F2 (Dentsply, Maillefer, Tulsa, OK, USA). Sodium hypochlorite was used as an irrigant to remove debris. Final irrigation was performed with ethylene diamine tetraacetic acid (EDTA) to make a smear-free layer using a Luer-lock needle for a period of 1 min.

### 2.7. Determination of Depth of Penetration of Liposomal CHX in Dentinal Tubules

Twenty-one teeth were selected for testing the depth of penetration. The following treatment was carried out for Group I and Group II:

Group I: Seven teeth were irrigated with 2% *w/v* CHX solution (added with 1% *w/v* rhodamine B dye);

Group II: Fourteen teeth were irrigated with liposomal CHX containing rhodamine B dye. (The rhodamine B-loaded liposomes were formulated similar to the CHX-loaded liposomes. The only difference was the addition of rhodamine B in place of CHX).

All the teeth were irrigated with 5 mL of respective irrigating solutions using 5 mL disposable syringe attached with a Luer-lock needle for a period of 1 min. The needle was maintained at 1 mm short of apex into the root canal and EndoActivator (Dentsply Sirona, Charlotte, NC, USA) was used to agitate. The same irrigation procedure was repeated two times. The volume of irrigation was 10 mL. All the teeth were washed with phosphate-buffered saline after the irrigation procedure. Each sample was sectioned into three parts, each of 1 mm thickness *viz.*, coronal third, middle third, and apical third with a low-speed diamond saw. Then, sections were viewed using the laser confocal scanning microscope (CLSM; LSM 980, Carl Zeiss, Germany) to evaluate the penetration depth of plain CHX as well as liposomal CHX. The depth of penetration of liposomal CHX and plain CHX solution into the dentin surface was detected by the fluorescence at 10× magnification. Excitation was performed at 543 nm to collect the emission emitted in the 560 nm process. The images captured were divided into four equal parts, and the depth of penetration at each section was measured. The mean values of the penetrated irrigants were recorded.

### 2.8. Cell Viability Assay

The cell viability assay of pure CHX and liposomal CHX was performed on two different types of mouse fibroblast cell lines *viz*., L929 and 3T3 cells. The cells (L929 and 3T3) were seeded in a 96-well plate at a density of 5 × 10^3^ cells/well with DMEM supplemented with fetal bovine serum (10%; Thermo Fisher Scientific, Waltham, MA, USA) and antibiotic solution (12%; HiMedia Laboratories, Mumbai). The cells were incubated with 100 µL of test substance at different concentrations of CHX in plain CHX solution as well as liposomal CHX ranging from 7.8 to 250 µg/mL. Plain liposomes, devoid of CHX, were also tested at the same range of concentrations. The untreated cells were used as the control for comparison. The treated L929 and 3T3 cells were incubated for 24 h. After the incubation period, the supernatant was removed and 100 µL of 0.5 mg/mL MTT (HiMedia, Mumbai, India) dissolved in HBSS solution was added to the plates. The cells were further incubated for 3 h at 37 °C. The supernatant was removed and 100 µL DMSO was added. The absorbance was calculated at 570 nm using a microplate reader (BioTek Instruments, Santa Clara, CA USA). The percentage cell viability was calculated using the formula as provided below and IC_50_ values were calculated by the GraphPad prism 9.0 software.

Cell viability (%)=(Test sample)/(control)×100

### 2.9. Antimicrobial Study

*Staphylococcus aureus*, *Fusobacterium nucleatum (F. nucleatum)*, and *Streptococcus mutans* of clinical strains were cultured. The cells were inoculated in brain heart infusion (BHI) broth and egg yolk agar for *F. nucleatum* at a concentration of 10^8^ cells/mL to develop a biofilm. Polystyrene 96-well plates were obtained, into which 100 µL of standard cell suspension was pipetted, and were incubated under anaerobic conditions for 72 h. Phosphate buffered saline was used to wash the biofilms and treated with a diluted series of liposomal CHX (equivalent to 2% *w/v* of plain CHX) and CHX solution (2% *w*/*v*) for 72 h before determining the minimal inhibitory concentration readings. Concurrently, planktonic bacterial cell suspension at 10^8^ cells/mL was used to obtain the MIC readings.

### 2.10. Statistical Analysis

Statistical analysis was analyzed by two-way ANOVA with Tukey’s post-hoc test (SPSS Software, version 23.0, SPSS INC. Chicago, IL, USA).

## 3. Results

### 3.1. Preparation and Characterization of Liposomal CHX

The liposomes were successfully prepared by the thin-film hydration technique. The particle size of liposomes was analyzed using the Malvern zeta sizer. The average liposomal particle size was found to be 148 ± 4.52 to 223 ± 3.63 nm. Batch 3, which was optimized, based on size, zeta potential, and drug encapsulation efficiency, was found to possess the average particle size of 178.40 ± 4.41 nm (Table 1). The polydispersity index value for all the batches was found below 0.4, indicating the homogeneity of the dispersion. The results of zeta potential of different batches of liposomes are shown in Table 1. The zeta potential values of all the batches prepared were found in the positive side; with 24.1 ± 2.60, 29.3 ± 2.42, 28.1 ± 2.56, 34.2 ± 2.60, and 37.8 ± 2.12 mV for Batches 1 to 5, respectively. The zeta potential of CHX liposomes was found in the positive side, owing to the fact that there may be hydrophobic interaction between chlorhexidine and HSPC along with the electrostatic interaction due to the charge interaction between the ketone group of HSPC and amine group of chlorhexidine, which might lead to the positive surface charge of liposomes [19,20,21,22].

**Figure 1:**
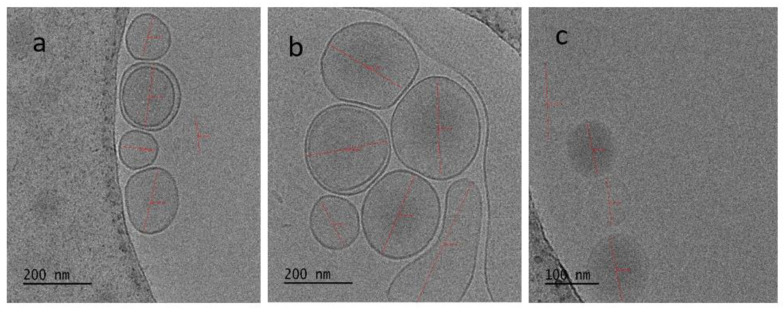
Cryo-electron microscopic images showing the surface morphology of CHX liposomes. (**a**,**b**) Cryo-EM images of multiple liposomes at 200 nm scale in which the lipid bilayer is also visible and (**c**) Cryo-EM image of liposomes at 100 nm scale.

The Cryo-EM images of optimized batch (Batch 3) of liposomes are shown in Figure 1. The vesicles were found to possess a spherical vesicular structure with a size of about 200 nm, which is inconsistent with the results obtained from Zeta Sizer analysis. Along with the almost spherical structure, the vesicles were found to be intact, discrete, and bilayered (Figure 1a,b). Cryo-EM image of liposomes at the magnification scale of 100 nm is shown in Figure 1c, which also showed the similar features as observed in Figure 1a,b.

**Figure 2:**
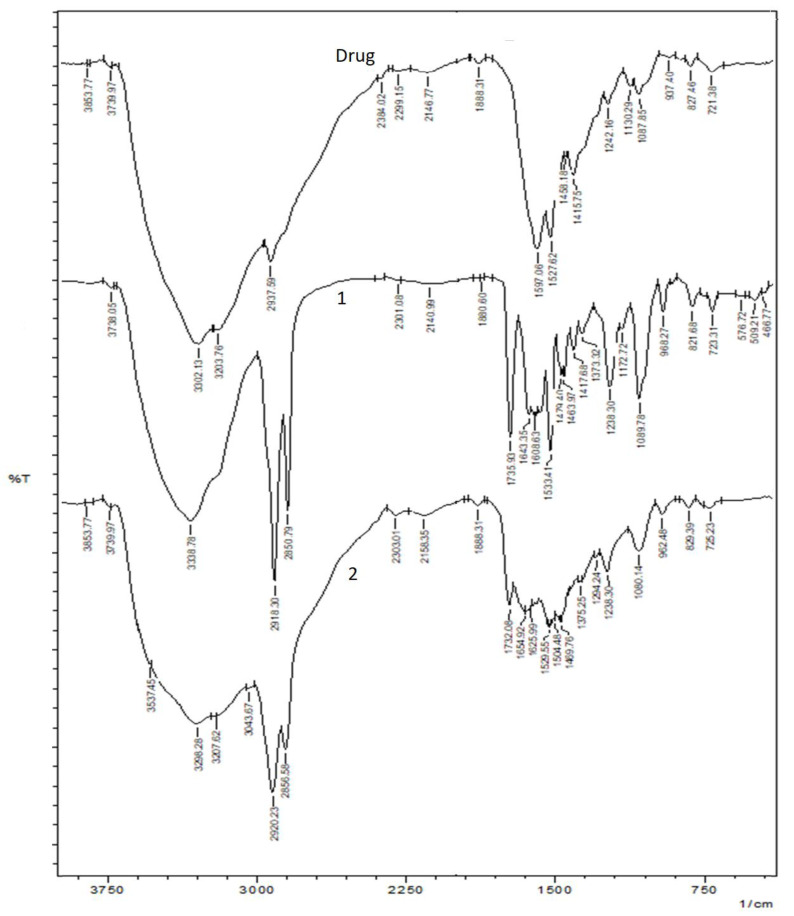
FTIR spectroscopic results. (Drug) Plain CHX, (**1**) physical mixture of CHX and excipients and, (**2**) CHX liposomal formulation.

The results of FTIR spectroscopy are shown in Figure 2. The FTIR data provide an idea of any chemical interaction between the drug and excipients. Figure 2(drug) shows the FTIR spectrum of the plain drug. The main stretching vibrations of 3300 to 3500 cm^−1^ for the N–H group describe the FTIR spectrum of CHX. The 2850 to 3000 cm^−1^ bands are the stretching vibration bands attributable to the aliphatic C–H group. There are also wavelength peaks of 1450 to 1550 cm^−1^ that can be assigned to the C group in the aromatic ring and ≈1238 to 1251 cm^−1^ that contribute to the stretching vibration frequency of the aliphatic amine group (C–N) [23] (Figure 2(drug)). These specific peaks were unaltered in the physical mixture of CHX and the excipient (Figure 2(1)) and liposomal formulation (Figure 2(2)), indicating the chemical compatibility between the drug and excipients.

### 3.2. In Vitro Drug Release Study

**Figure 3:**
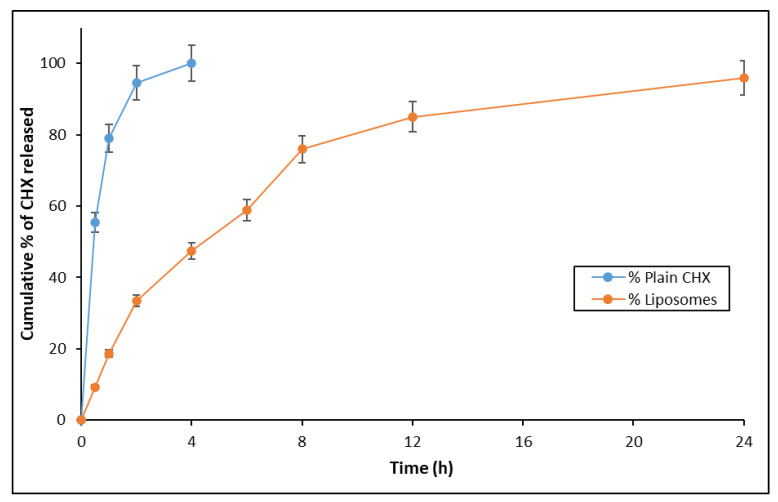
In vitro drug release profile of CHX in its plain form and liposomal form.

The in vitro drug release study was carried out for the plain 2% CHX solution as well as the CHX-loaded liposomal formulation (Figure 3). The liposomal formulation showed a sustained release of drug up to 24 h, whereas the plain CHX solution showed 100% drug release at 4 h. Herein, the 24 h drug release is not relevant to the root canal of the teeth. The authors are working extensively on dental products and, thus, the authors are analyzing the prospect of using the liposomal formulation of extended release in the case that it can be used as long-acting dental inserts. The purpose of analyzing the drug release for 24 h was with respect to the hold time of drug for these products.

### 3.3. Evaluation of Liposomal CHX with Respect to Depth of Penetration in Dentinal Tubules

**Figure 4:**
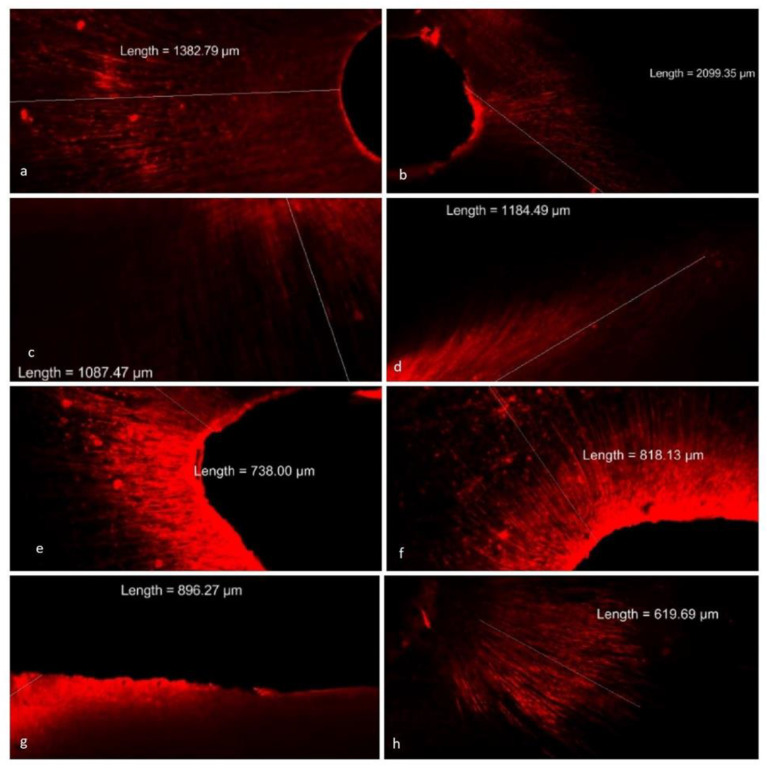
Confocal images depicting the depth of penetration of liposomal CHX containing rhodamine B dye into dentinal tubules. Photographs (**a**,**b**) depict the penetration of liposomal CHX into dentinal tubules in coronal third. Photographs (**c**,**d**) show liposomal CHX penetrating in middle third. (**e**,**f**) Liposomal CHX average penetration in apical third was less when compared with other thirds. (**g**,**h**) Images of penetration in the coronal and apical third, respectively of normal CHX. The penetration was significantly less as compared with liposomal CHX.

The results of depth of penetration of plain CHX solution and liposomal CHX into dentinal tubules are shown in Table 2 and Figure 4. Liposomal CHX showed significantly (*p* < 0.001) better depth of penetration in all the regions of dentinal tubules in comparison with the plain CHX solution. With liposomal CHX, the depth of penetration is significantly (*p* < 0.001) higher in coronal (1549.91 ± 422.56 µm) compared with middle (1115.68 ± 410.50 µm) and apical (758.34 ± 93.46 µm). The depth of penetration observed with middle and apical thirds was also significantly (*p* < 0.001) different from each other. When compared between the two groups, the normal CHX group exhibited a significantly (*p* < 0.001) lower depth of penetration compared with the liposomal CHX group in all the regions with the value of the impact of intervention as 41.6% (Table 3). The depth of penetration is with respect to the rhodamine B dye, and it does not reflect the rate of release of chlorhexidine. The depth of penetration is a property of liposomes and, thus, the results provided in Table 2 and Figure 4 complement each other.

### 3.4. Antimicrobial Assay

The viable bacterium remaining after exposure to plain CHX solution was considerably more than observed with liposomal CHX. This indicates that liposomal CHX eliminated more bacteria compared with the plain CHX solution. Liposomal CHX decreased bacterial load after 24 h to a greater extent than the plain CHX. The colony counts of bacterium after 72 h of exposure to liposomal CHX were found to be zero (Table 4).

### 3.5. Cell Viability Assay

The cytotoxicity of plain CHX solution and liposomal CHX against L929 and 3T3 mouse fibroblast cells was determined by the MTT assay. The pure CHX solution exhibited an IC_50_ value of 12.32 ± 3.65 µg/mL in L929 cell line and 29.07 ± 2.14 µg/mL in 3T3 cell line. Liposomal CHX exhibited an IC_50_ value of 37.90 ± 1.05 µg/mL and 85.24 ± 3.22 µg/mL in L929 and 3T3 cell lines, respectively. The percentage of cytotoxicity was less in liposomal CHX compared with the pure CHX solution (Table 5). The results showed approximately 3-fold decreased cytotoxicity in liposomal CHX as compared with the plain CHX in both cell lines. The obtained results were further confirmed by observing the cellular morphology under the brightfield microscope after different treatments. The brightfield microscopic images are given in Supplemented Material (II. Brightfield microscopic images; Figures S4–S7). As shown in these figures, the cells were dead even at the lower concentration of plain CHX solution; however, the cells retained their integrity after various treatments of liposomal CHX. Blank liposomes (without CHX) did not show any cytotoxicity. More than 80% of both cells were alive even at the highest tested concentration (250 µg/mL).

## 4. Discussion

Nanoencapsulation of CHX has been shown to increase the action of the drug in previous reports. In this study, the preparation of CHX-loaded liposomes was achieved using the thin-film hydration method. In this experiment, an attempt was made to develop liposomal CHX, which has scarcely been reported for the study of the depth of penetration of the teeth using rhodamine B dye with a confocal laser scanning microscope (CLSM). The liposomal formulation had good penetration when compared with the plain CHX. The application of nanotechnology approaches for effective delivery of CHX has been reported in a few of the previous reports. In a previous study, mesoporous silica nanoparticles of CHX were studied for antibacterial effects. Mesoporous silica nanoparticles were effectively loaded with CHX and its release from the nano-CHX was confirmed [20]. Based on the positive results derivation, it could be assumed that the CHX nanoformulation may have better penetrating efficiency in dentin, which may subsequently eradicate oral biofilms and control the bacteria. The hydrophobic components of liposomes are repelled by water molecules leading to liposome self-assembly. Additionally, phosphatidylcholine (PC) and dipalmitoyl PC can be used for liposome generation [7].

In another study, encapsulation of CHX was achieved by loading CHX inside the polymeric self-assembled tri-layered nanoparticles (TNPs). The TNPs improved the physico-chemical equilibrium of nanoparticles without the use of additional surfactants in the aqueous mixture solution. These TNPs proved to efficiently encapsulate CHX for the targeted drug delivery of dentinal matrix, which was attempted to be used in disinfection of the root canal [18].

The colloidal system’s stability is assessed by zeta potential. It is a measure of the repulsive forces that exist between the particles. Particles with stronger repulsive forces are less likely to combine and are stable. The surface charge value of the particles reflects the stability of the nanosuspensions, given that vesicular dispersions with higher ZP values are electrostatically stabilized nanosuspensions [23]. In the present study, liposomes display a considerably higher potential zeta value suggesting strong physical stability. In addition, a high positive charge is beneficial in the permeation of cell [24]. When the concentration of CHX was increased from 10 to 50 mg, the zeta potential values were found to increase. Particles with stronger repulsive forces are less likely to combine and are stable.

Although Batch 5 with 50 mg of CHX showed the highest zeta potential value, Batch 3 was considered as an optimized batch, based on the particle size, entrapment efficiency, and zeta potential. In this study, the drug encapsulation efficiency (%EE) was found to be influenced by the total amount of CHX incorporated in the liposomes. The EE values were found between 36 ± 2.42% and 76 ± 2.80%. Batch 3 showed the highest EE% value of 76 ± 2.80%. Moreover, the percentage of EE increased considerably when the CHX concentration increased from 10 to 30 mg. However, further increase in the amount of CHX to 40 mg led to a reduction in the percentage of EE, which may be due to the vesicle leakage of CHX [25].

To check the visibility of lipidic bilayers, the vesicles were suitably focused in Cryo-EM. The lipidic bilayers of the liposomes were clearly visible in this image (Figure 1). In the FTIR spectra, the peaks of absorbance indicate different functional groups. Specific bond types and, thus, specific functional groups absorb different wavelengths of infrared radiation, as explained above in the “Results” section.

Nanoencapsulation could result in a sustained drug release and this prolonged delivery period may lead to a complete elimination of bacteria in dentinal tissues [18,26]. The in vitro diffusion study demonstrated the sustained release of CHX from the liposome compared with the pure CHX. The release of CHX from liposomes was nearly 100% in 24 h; while 100% CHX release was obtained for pure drug within 4 h. The obtained results provide insight that CHX-loaded liposomes can act as a reservoir for the long-term release of drug after they were located inside dentinal tubules. Moreover, the above results infer that liposomal CHX can demonstrate a long-term anti-microbial action compared with the pure solution. Optimal ratio of lipid may help in further sustaining the release of drug leading to a decrease in usage frequency, which will be convenient to patients in comparison with the normal CHX solution.

The drug release kinetics were deduced from the in vitro drug release data by subjecting the date with respect to zero-order equation, first-order equation, and Higuchi’s equation [27]. For the zero-order pattern, the equation was found to be y = 3.9041x + 22.234 with R2 value of 0.7655. For the first-order release pattern, the equation was y = 0.0338x + 1.3825 with R2 value of 0.5562. However, for Higuchi’s release pattern, the equation was found to be y = 22.449x with R2 value of 0.8482. Therefore, the drug release pattern from liposomes was found to be diffusion dominated, according to Higuchi’s equation.

The teeth were treated with 2% *w/v* CHX solution (added with 1% *w/v* rhodamine B dye) or with liposomal CHX containing rhodamine B dye. The results indicated that the depth of penetration of normal CHX solution is higher in coronal (700 ± 75 µm) compared with the middle third (623 ± 68 µm) (*p* > 0.001) and apical third (331 ± 39.40 µm) (*p* < 0.001). However, the depth of penetration was not significantly different between the coronal and middle third of the roots with respect to normal CHX. This may be due to the fact that the tubule diameter is almost similar to the coronal and middle thirds and the agitation of EndoActivator might have helped in penetrating the above areas compared with the middle third.

Rhodamine B dye was mixed with both liposomal CHX as well as normal CHX irrigating solutions at 1% concentration to evaluate the depth of penetration [28]. The penetration of irrigant is essential to eradicate the microorganisms. *E. faecalis* can penetrate deep into dentinal tubules and co-aggregate with other organisms [29]. Dentinal tubules may be invaded by bacteria, where they are unaffected by instrumentation and irrigation. These procedures are effective only on the surface of the canal [11]. Endodontic infection caused by bacteria that have colonized the dentinal tubules might cause failure in treatment. According to Zou et al. (2010), the microorganisms can produce endotoxin that can penetrate up to 500 µm into the root dentin. The failure in endodontic treatment may be attributed to the deep penetration of bacteria into dentinal tubules and also the buffering capacity of dentin which protects the bacteria from the CHX effect [30,31].

The systems commonly used as irrigant activation techniques include sonic, ultrasonic agitation, manual activation with gutta-percha cones, and agitation with brushes [11]. In a study by Kanumuru et al. (2015), it was shown that for the effective penetration of irrigants up to a working length and into the lateral canals, this can be achieved by the irrigant activation using reciprocation movement [10]. In the previous study, the mean penetration depth values observed with 2% CHX solution in coronal, middle, and apical thirds were 138, 80, and 44 µm, respectively for the conventional syringe group; whereas the mean penetration depth values in passive ultrasonic irrigation were 209, 138, and 72 µm, respectively in coronal, middle, and apical thirds [11,28]. In another study, it was proved that the irrigant penetrated to a depth of 249.9 µm in coronal third, 163 µm in middle third, and 42 µm in apical third [30]. In contrast, the CHX solution used in the present study showed comparatively higher values for depth of penetration. All the previous reports and the results of the present study indicated high variability in the data of depth of penetration. This high variability may be attributed to several factors. Dentin variables, such as depth, type, and caries will affect the size and patency of dentinal tubules [11,32]. In addition, temperature and moisture may also influence penetration [33]. Moreover, the application of EndoActivator could have influenced the depth of penetration [34].

Herein, Cyro-EM was used to study the morphology of the liposomes. The liposomes were nearly spherical in shape. Bilayered vesicular structure, which is a prominent feature of liposome, was observed in Cryo-EM. The freeze fracture electron microscope is considered the ideal method for the characterization of liposomes [35]. Liposomal CHX was able to penetrate deep inside the tubules compared with normal CHX. This may be due to the reduced particle size. Moreover, the spherical nature of liposomes can be the main cause of the increase in penetration [36]. In another study, the diode laser-assisted irrigant activation technique showed better penetration depth in all of the three aspects of root dentin [34]. Furthermore, the presence or absence of smear layer determines the depth. In this study, EDTA was used to remove the smear layer during the biomechanical preparation of the tooth.

Our study is in agreement with previous studies, where the depth of penetration is more in coronal third followed by middle and apical thirds [30]. Animal studies have proven that the liposomes may have the ability to enhance or decrease the penetration [37,38]. CLSM was used in our study as the sample preparation is simpler, produces lower artefacts, and provides accurate results [34]. According to Galler et al. (2019), sodium hypochlorite and EDTA showed median penetration depths of 700–900 μm [39].

Previous studies have shown that tubular density diminishes toward the apex, and due to this penetration ability, it is reduced. As the teeth selected were of the lower age group who opted for an orthodontic treatment, the chance of sclerosis is unlikely. EndoActivator was used here as it has shown better efficacy in irrigation than the conventional needle [39]. This might have enhanced the dentinal penetration. However, one of the problems with liposomes is physical instability, which may lead to leakage of CHX from the lipid vesicles during storage and in vivo administration. However, this can be addressed with an optimized composition of lipids and excipients.

Microorganisms are the major cause for treatment failure in root canals. The bacterium has the ability to invade inside the dentinal tubules and, thus, co-aggregates with each other [32]. Antimicrobial analysis revealed the reduction in bacterial counts of *Staphylococcal aureus* and *Streptococcus mutans. Streptococcus mutans* was selected due to the fact that it has been proven to be persisting in symptomatic and asymptomatic apical periodontitis. *Staphylococcus aureus* is one of the commonly isolated organisms from re-treatment cases. *Fusobacerium nucleatum* is the organism which is generally observed in primary endodontic infections [40,41]. Liposomal CHX was effective in reducing the bacterial counts after 72 h. In this study, as liposomal CHX has penetrated deep into dentinal tubules and as it reduced the bacterial counts, this drug may be effective in eradicating bacteria within the root canals and oral cavity when used as a disinfectant.

The cell viability and half-maximal inhibitory concentration (IC_50_) of plain CHX solution and liposomal CHX were performed in L929 and 3T3 mouse fibroblasts by the MTT assay. This study was performed due to the fact that CHX is commonly used as a cavity disinfectant and, thus, may penetrate dentin and come in contact with pulpal fibroblasts. CHX as a cavity disinfectant may inhibit the MMPs and result in an increase in the life span of composite restoration [16]. In a previous study, the highest toxicity of 2% CHX was seen at a concentration of 0.016% at an interval of 72 h [40]. Toxic potency of CHX depends on the composition of exposure media. Exposure dose and duration of exposure are shown by the results of in vitro studies on cytotoxicity of CHX against human gingival cells [41]. Our data exhibited dose-dependent toxicity of both plain CHX solution and liposomal CHX. However, considerably reduced cytotoxicity is observed in the liposomal form at 24 h. Arbitrarily, since 2 mL of irrigant is used as a cavity disinfectant in tooth, the liposomal CHX may be considered due to its decreased cytotoxicity.

While the liposomes of the present study show utility in cell killing simply by delivering the drug cargo to the cell interior, other drug formulations require the release of the drug directly at the subcellular site/organelle of action (e.g., nucleus, mitochondria, lysosome, endoplasmic reticulum, Golgi body). Although cell surface ligands are effective for active targeting as well as in improving the drug efficacy, this is not always enough to deliver the drugs to specific subcellular locations. This aspect has prompted further efforts to develop more sophisticated nanosystems [42,43].

The stability of these liposomes at different storage conditions is not assessed in this study. However, based on our previous studies, it can be presumed that the prepared liposomes could be stable for 6 months at 5 ± 3 °C. Nevertheless, a detailed stability study of these liposomes has to be performed to determine the shelf life.

Although encouraging results have been obtained with liposomal CHX in the present study, the major limitation is that the antimicrobial efficacy of the liposomal formulation could have been tested within the root canal of the extracted tooth. However, a future perspective of this study is to carry out a detailed toxicity assessment of this formulation and then perform a clinical study to appraise the results obtained in this preliminary study.

## 5. Conclusions

In the present work, liposomal formulation of CHX demonstrated enhanced penetration into dentinal tubules as well as better antimicrobial activity compared with CHX alone. This provides insight into the potential of liposomal formulations and development of delivery system for dental infections. Further optimization of this system can prove to be quite beneficial in the development of dental disinfection with prolonged therapeutic activity. Owing to the recent advancement in drug delivery for root canal infection, nanotechnology proved to be a potential platform for the efficient treatment of these infections. Although CHX has widely been considered as a standard drug for endodontic applications, microorganisms are generally observed even after its use, and it has been attributed to a lack of drug penetrating efficiency into dentinal tubules. Fabrication of liposomes is one of the approaches to improve the efficacy of CHX and to overcome the drawbacks.

## Figures and Tables

**Table 1:** Composition of different batches of liposomes prepared and results of encapsulation efficiency, size, and zeta potential.

Batches	Cholesterol (mg)	HSPC (mg)	Drug (mg)	Encapsulation Efficiency (%)	Size (nm)	Zeta Potential (mV)
1	90	10	10	36 ± 2.42	148.2 ± 4.52	24.1 ± 2.60
2	90	10	20	48 ± 1.76	156.0 ± 5.36	29.3 ± 2.42
3	90	10	30	76 ± 2.80	178.4 ± 4.41	28.1 ± 2.56
4	90	10	40	56 ± 2.67	206.4 ± 3.63	34.2 ± 2.60
5	90	10	50	52 ± 2.45	223.6 ± 3.63	37.8 ± 2.12

The results are presented as mean ± SD, *n* = 3. HSPC: Hydrogenated soy-phosphatidylcholine; mg: Milligram; mV: Millivolts.

**Table 2:** Depth of penetration of plain CHX solution and liposomal CHX in dentinal tubules.

Groups	Sections	Number of Samples	Depth of Penetration (µm)	95% Confidence Interval for Mean	
				Lower Bound	Upper Bound
Group I (Plain CHX solution containing Rhodamine B)	Coronal	7	700.67 ± 75.78	630.58	770.75
	Middle	7	623.83 ± 68.48	560.49	687.16
	Apical	7	331.74 ± 39.40	295.30	368.18
	Total	21	552.08 ± 173.54	473.08	631.07
Group II (Liposomal CHX containing Rhodamine B)	Coronal	14	1549.91 ± 422.56	1305.93	1793.89
	Middle	14	1115.68 ± 410.50	878.66	1352.70
	Apical	14	758.34 ± 93.46	704.38	812.30
	Total	42	1141.31 ± 469.18	995.10	1287.52

The results are presented as mean ± SD, *n* = 3. CHX: Chlorhexidine digluconate; µm: Micrometer.

**Table 3:** Two-way ANOVA results of depth of penetration of plain CHX solution and liposomal CHX in dentinal tubules.

Source	ANOVA F-Value	*p*-Values	Effect Size
Main effect due to Group	58.982	0.000	0.509
Main effect due to Subgroup	19.156	0.000	0.402
Interaction effect of Group vs. Subgroup	2.932	0.061	0.093

CHX: Chlorhexidine digluconate; ANOVA: Analysis of variance.

**Table 4:** Viable bacterial cells and colony forming units remaining after irrigation with liposomal CHX and 2% plain CHX.

Viable Bacteria/Colony Counts	Organism	Liposomal CHX	2% CHX
Viable bacterium remaining after exposure to the drug for 12 h (µg/mL)	*Staphylococcus aureus*	˃16	˃32
	*Streptococcus mutans*	˃4	˃8
	*Fusobacterium nucleatum*	>4	>16
Colony counts of bacterium after 72 h of exposure (‘-‘ = No colonies)	*Staphylococcus aureus*	-	>2
	*Streptococcus mutans*	-	>2
	*Fusobacterium nucleatum*	-	-

The results presented are the values from three replicates. CHX: Chlorhexidine digluconate.

**Table 5:** Results of cell viability assay of plain CHX solution and liposomal CHX.

Samples	Test Conc. (μg/mL)	Cell Viability (%)		IC_50_ (μg/mL)	
		L929 Cells	3T3 Cells	L929 Cells	3T3 Cells
Plain CHX solution	250	10.41 ± 0.30	16.93 ± 0.13	12.32 ± 3.65	29.07 ± 2.14
	125	10.41 ± 0.11	18.94± 1.23		
	62.5	10.46 ± 0.23	36.22 ± 0.44		
	31.25	12.10 ± 0.39	43.04 ± 0.36		
	15.62	47.95 ± 0.49	61.47 ± 0.11		
	7.81	72.88 ± 0.30	76.31 ± 0.98		
Liposomal CHX	250	10.05 ± 0.17	26.98 ±0.76	37.90 ± 1.05	85.24 ± 3.22
	125	10.18 ± 0.39	43.32 ± 1.28		
	62.5	10.26 ± 0.09	64.05 ± 0.03		
	31.25	69.04 ± 0.56	69.53 ± 0.48		
	15.62	89.59 ± 0.22	75.99 ± 1.11		
	7.81	95.34 ± 1.65	82.67 ± 0.73		

The results are presented as mean ± SD, *n* = 3. **CHX**: Chlorhexidine digluconate; IC_50_: Half-maximal inhibitory concentration.

## Data Availability

Not applicable.

## Supplementary Materials

The following supporting information can be downloaded at: https://www.mdpi.com/article/10.3390/nano12193372/s1. Figure S1. UV spectrum of CHX solution in water recorded using UV/ Vis Spectrophotometer; Figure S2. UV spectrum of blank liposomes (containing only the excipients of liposomes without CHX) recorded using UV/ Vis Spectrophotometer; Figure S3. UV spectrum of CHX liposomes recorded using UV/ Vis Spectrophotometer; Figure S4. The bright-field microscopic images of L929 cells at 24 h after different treatments of plain CHX solution; Figure S5. The bright-field microscopic images of L929 cells at 24 h after different treatments of liposomal CHX; Figure S6. The bright-field microscopic images of 3T3 cells at 24 h after different treatments of plain CHX solution; Figure S7. The bright-field microscopic images of 3T3 cells at 24 h after different treatments of liposomal CHX.
