# Supplementary material for: Development of Lipidic Nanoplatform for Intra-Oral Delivery of Chlorhexidine: Characterization, Biocompatibility, and Assessment of Depth of Penetration in Extracted Human Teeth
Source: Nanomaterials (Basel). 2022 Sep 27;12(19):3372. doi: 10.3390/nano12193372 (PMC9565570; doi:10.3390/nano12193372)
Supplement: Supplementary file 1 [file nanomaterials-12-03372-s001.zip › nanomaterials-1817768-supplementary.pdf]

SUPPLEMENTED MATERIAL

Development of Lipidic Nanoplatform for Intra-Oral Delivery of Chlorhexidine: Characterization, Biocompatibility and Assessment of Depth of Penetration in Extracted Human Teeth

Krishnaraj Somyaji Shirur [1], Bharath Singh Padya [2], Abhijeet Pandey [2], Manasa Manjunath Hegde [3], Aparna I Narayan [4], Bola Sadashiva Satish Rao [3], Varadaraj G Bhat [5], and Srinivas Mutalik [2,*]

I) Selection of 254 nm as λmax for the estimation of CHX

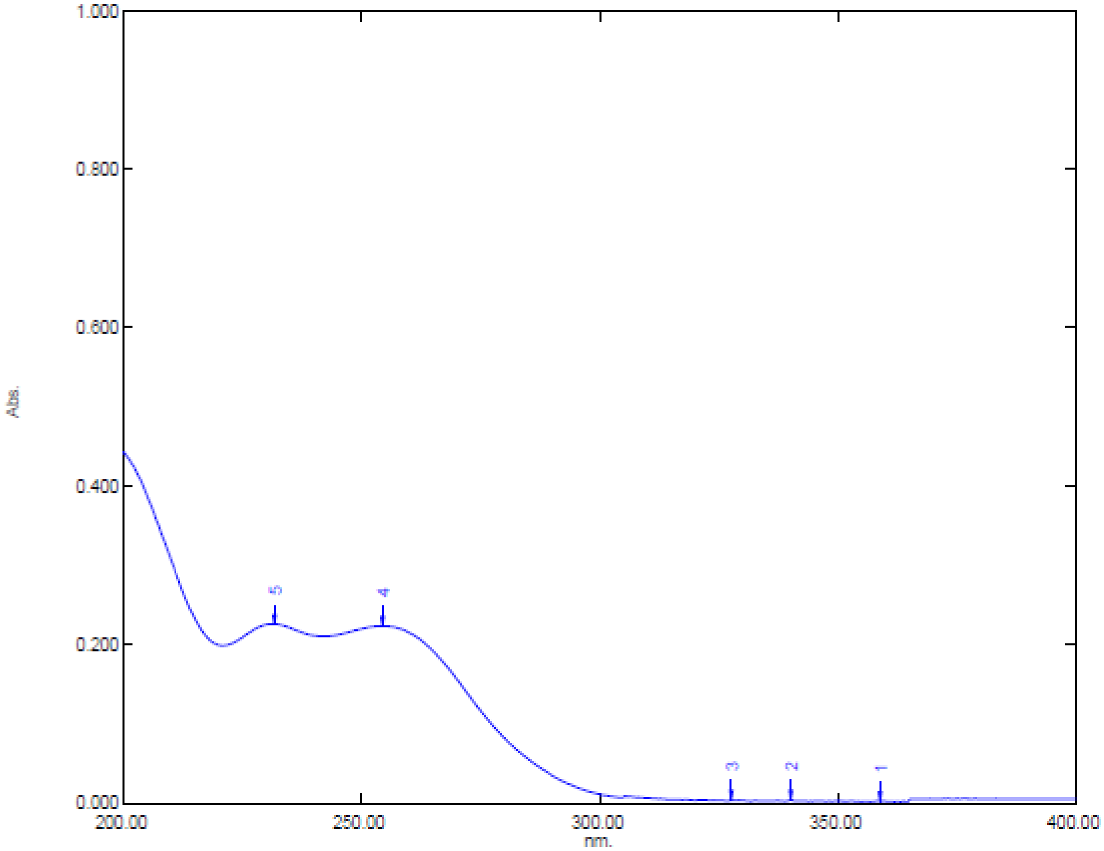

Figure S1. UV spectrum of CHX solution in water recorded using UV/ Vis Spectrophotometer

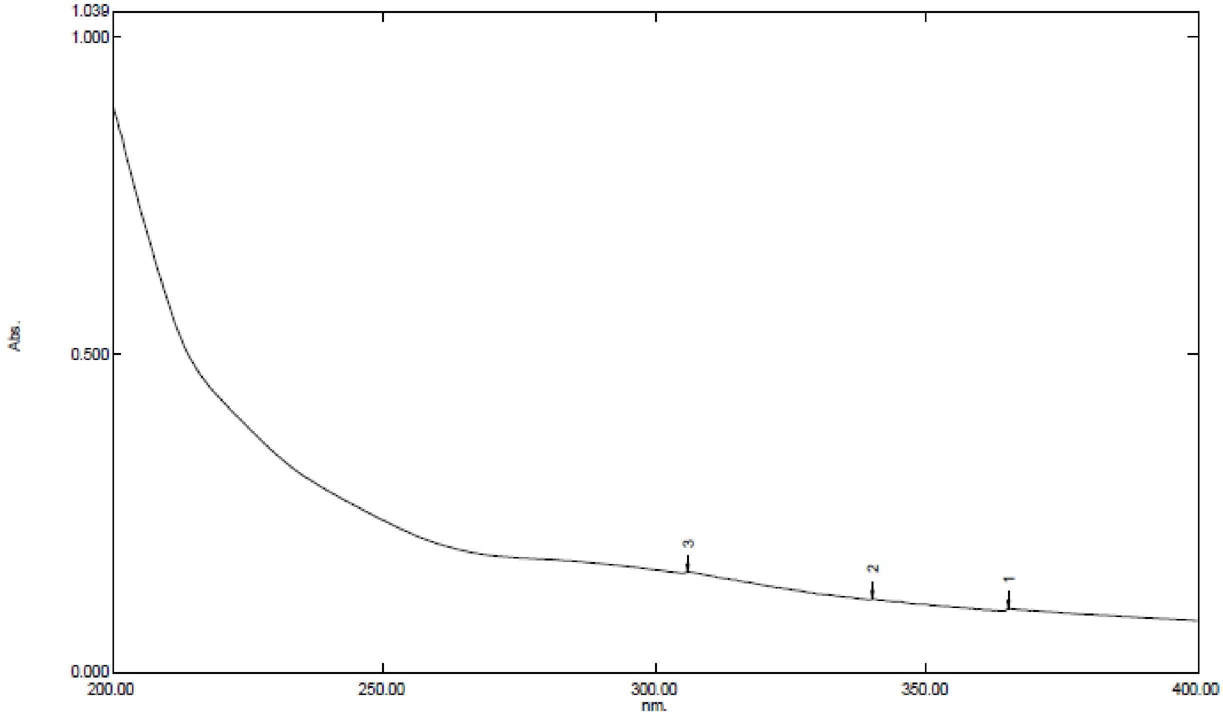

Figure S2. UV spectrum of blank liposomes (containing only the excipients of liposomes without CHX) recorded using UV/ Vis Spectrophotometer

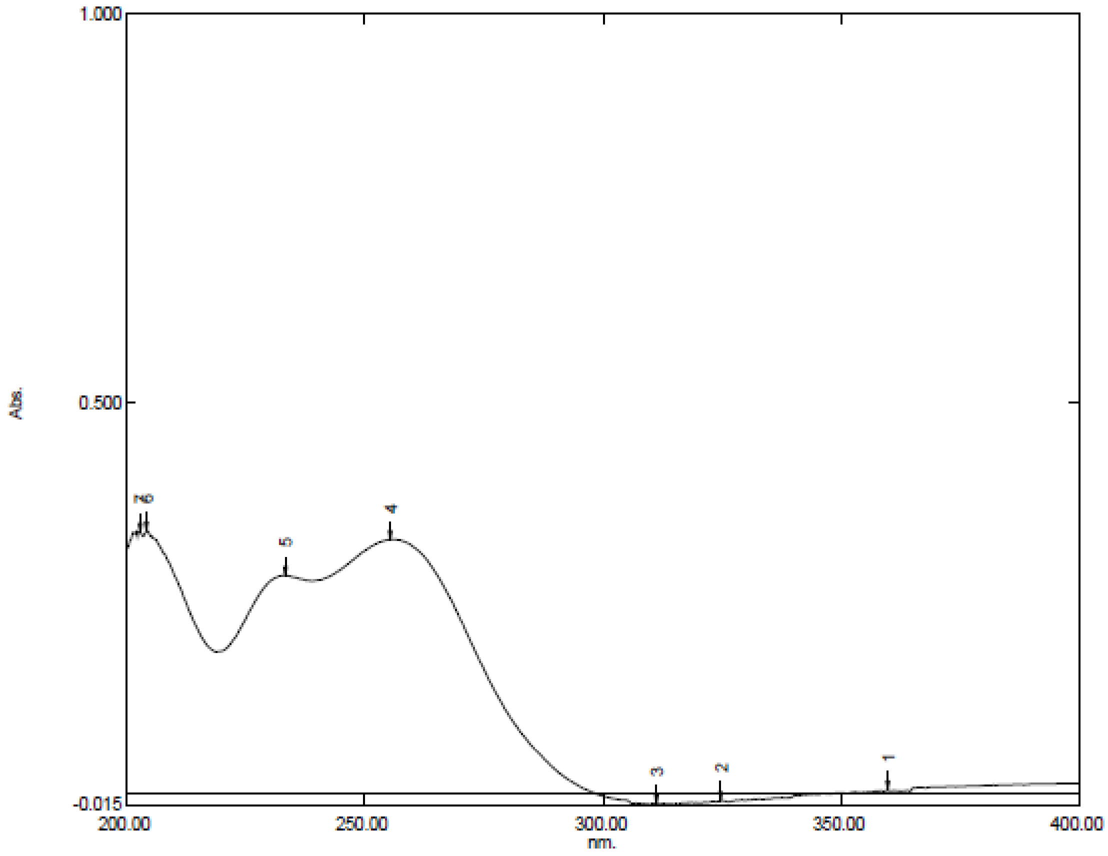

Figure S3. UV spectrum of CHX liposomes recorded using UV/ Vis Spectrophotometer

II) Bright-field Microscopic Images: The fibroblast cells, L929 and 3T3, were treated with different concentrations of plain CHX solution and liposomal CHX for 24 h. After incubation, the cells were observed under Bright-field microscope to study the cell morphology. The images were captured at the magnification of 10x/ 20x and represented as shown below.

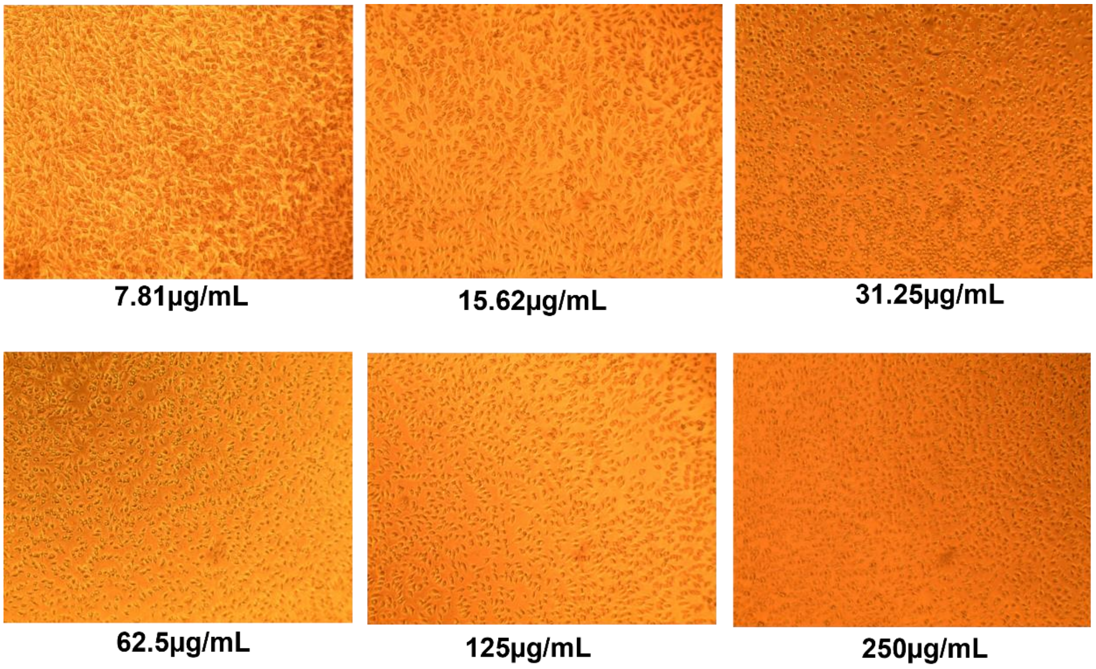

Figure S4. The bright-field microscopic images of L929 cells at 24 h after different treatments of plain CHX solution

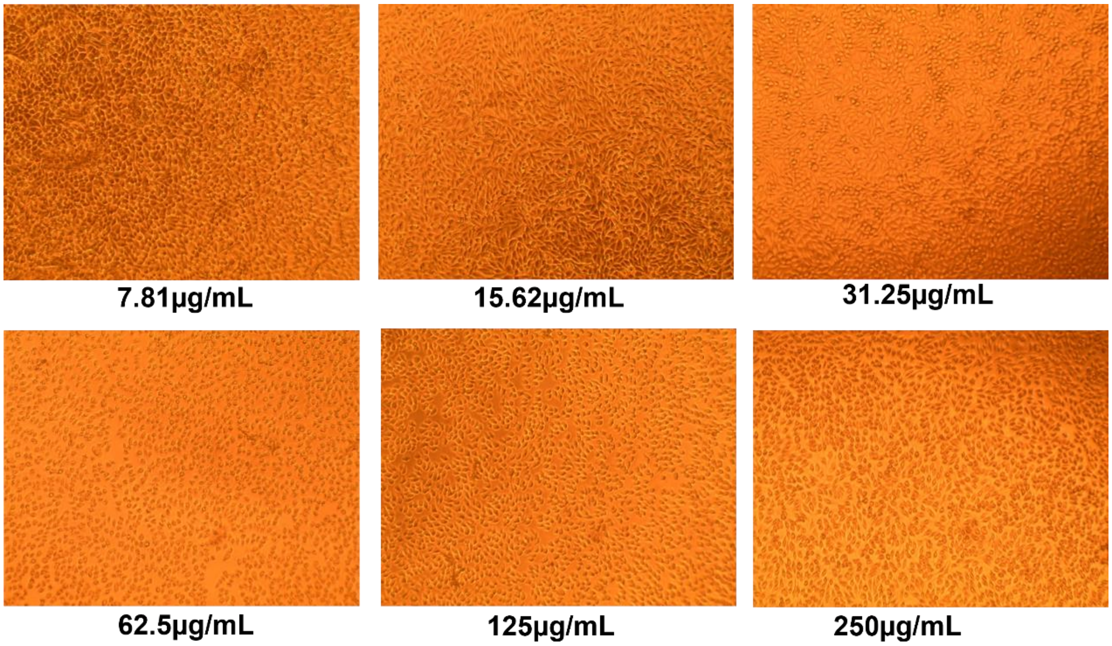

Figure S5. The bright-field microscopic images of L929 cells at 24 h after different treatments of liposomal CHX

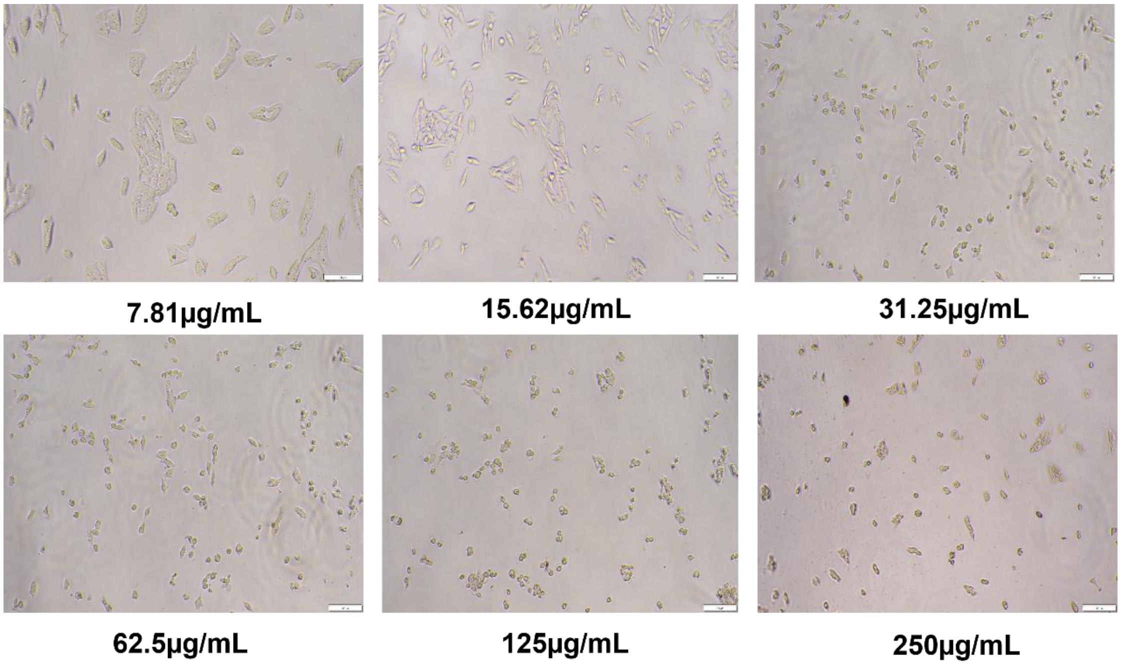

Figure S6. The bright-field microscopic images of 3T3 cells at 24 h after different treatments of plain CHX solution

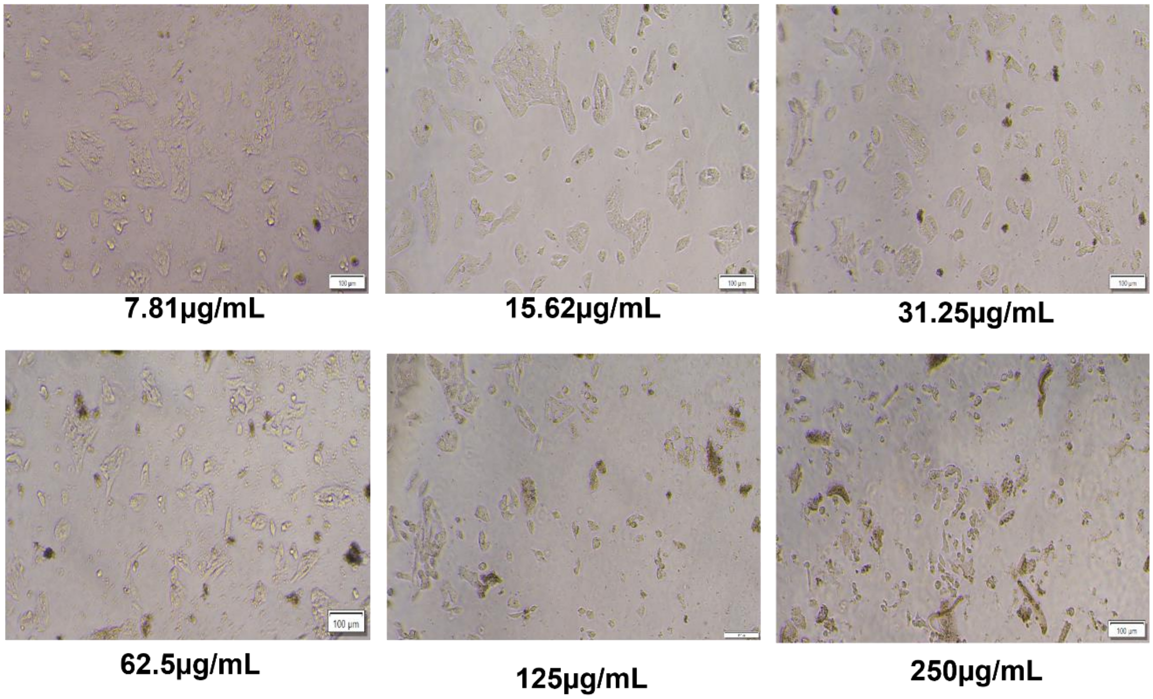

Figure S7. The bright-field microscopic images of 3T3 cells at 24 h after different treatments of liposomal CHX
